# Ustekinumab in the Treatment of Psoriasis and Psoriatic Arthritis

**DOI:** 10.1007/s40744-015-0010-2

**Published:** 2015-03-17

**Authors:** Laura J. Savage, Miriam Wittmann, Dennis McGonagle, Philip S. Helliwell

**Affiliations:** 1 Leeds Institute of Rheumatic and Musculoskeletal Medicine, University of Leeds, Leeds, UK; 2 NIHR Leeds Musculoskeletal Biomedical Research Unit, Chapel Allerton Hospital, Leeds, UK; 3 Centre for Skin Sciences, School of Life Sciences, University of Bradford, Bradford, UK

**Keywords:** IL-12/IL-23 inhibition, Psoriasis, Psoriatic arthritis, Treatment, Ustekinumab

## Abstract

Biologics have revolutionized the therapy of the psoriatic disease spectrum. These new classes of drugs also allow deeper insight into the pathogenesis of the disease and highlight the existence of distinct “molecular” disease subgroups as evidenced by the spectrum of clinical response seen. Molecules associated with both the interleukin (IL)-17 and interferon (IFN)γ pathways have important functions in psoriatic inflammation, and both are targeted by drugs acting on the p40 subunit shared by IL-12 and IL-23. These IL-12 family members are upstream of pathways characterized by the production of IFNγ and IL-17 related molecules, including IL-17, IL-22, and CCL20. We here summarize the mode of action and clinical studies of the p40 inhibitor ustekinumab with focus on both psoriasis and psoriatic arthritis.

**Electronic supplementary material:**

The online version of this article (doi:10.1007/s40744-015-0010-2) contains supplementary material, which is available to authorized users.

## Introduction

The pathogenesis of the psoriatic disease spectrum involves a plethora of cells and mediators. Given the complexity of the skin cytokine network, the cross regulation between infiltrating leukocyte subsets, endothelial cells and tissue resident mesenchymal and epithelial cells as well as tissue resident immune cells including innate leukocyte cell (ILC) subsets, it seems surprising that disease symptoms are responsive to a relatively wide array of interventions. In particular, patients with psoriasis respond to treatment interfering with lymphocyte activation, the tumor necrosis factor (TNF) pathway, agents blocking interleukin (IL)-17 or the IL-12/23p40 subunit.

The reason for different pathway blockage to work therapeutically in psoriasis lies most probably in their synergistic action which ultimately drives psoriatic inflammation. Synergistic activity regarding cell activation and cytokine production has been highlighted in a large number of studies mainly based on in vitro work. Very potent “mediator combinations” which can cause significant activation and proliferation/differentiation responses in the skin compartment are, for example, TNF + IL-17, IFNγ + TNF, and IFNγ + IL-17 [1]. Enhanced pro-inflammatory properties have also been described for the combined action of IL-22, IL-17, TNF, and IFNγ both in the skin and synovial compartment. The p40 unit shared by IL-12 and IL-23 is a fascinating therapeutic target as it influences two important effector cytokines, IFNγ and IL-17, the production of which is regulated by IL-12 and IL-23, respectively. Ustekinumab (UST) is a monoclonal antibody which targets the p40 subunit shared by IL-23 and IL-12.

## The Role of IL-12/23 in Inflammatory Immune Responses

The p40 β-chain can pair with the p35 or p19 subunit to form the heterodimeric cytokine IL-12 or IL-23, respectively. IL-12 and IL-23 are members of the IL-12 family along with IL-27 and IL-35 (for a review see [2] ). The p35 subunit of IL-12 is expressed ubiquitously whereas p40 expression largely restricted to antigen presenting cell (APC) types. Although p40 homodimers have been described for their antagonistic action on IL-12/IL-23, this has not been convincingly shown in the human system. The p19 and p35 on their own are biologically inactive. IL-12 and IL-23 each bind to a two-subunit receptor complex. They share the IL-12Rβ1 receptor but differ regarding signaling pathway activation by binding to the high affinity IL12Rβ2, which is highly expressed on type 1 cells (including T helper (Th) cells type 1 and ILC1) and IL-23R, respectively. IL-23R is one of the susceptibility genes highlighted by genome-wide association studies for both psoriasis and psoriatic arthritis (PsA) as is IL12B, which encodes for p40 [3]. IL-23 and IL-12 can both activate molecules of the same signaling pathways and these include JAK2, TYK2, STAT1, STAT3, STAT4, and STAT5. However, IL-12 predominantly signals via STAT4 phosphorylation, whereas IL-23 has a stronger impact on STAT3 pathway activation. There is a positive feedback loop in that STAT3 pathway activators (e.g., IL-23, IL-6, OSM, IL-22) can upregulate the cell surface expression of IL-23R; similarly STAT4 activation leads directly and indirectly via IFNγ secretion on IL-12Rβ2 expression [4]. Importantly high expression of IL-12Rβ2 is also influenced by IL-18 and by type I IFNs, which are highly expressed in psoriatic inflammation.

IL-12, which is mainly produced by macrophages and dendritic cells (DC), is a crucial molecule for polarization of CD4+ cells along the Th1 lineage [5, 6]. It is also well described for its action on cytotoxic CD8+ T lymphocytes and natural killer (NK) cells which ultimately can lead to their enhanced cytotoxicity. By acting on NK, CTL, Th1, and ILC type 1 the presence of IL-12 will lead to production of IFNγ. The action of IL-12, in particular regarding IFNγ production, can be supported by co-stimulators and among the soluble factors TNFα as well as IL-18 are known for this action. IFNγ and IL-12 act in a positive feedback loop in a number of ways. IL-12 induced IFNγ and IFNγ primes APCs for IL-12 production. IFNγ is a prototypic “priming” signal which means it makes cells (macrophages, DCs, keratinocytes) much more susceptible to any “second” signal such as TNFα. In the context of skin inflammation, and in particular psoriasis, it is of interest that IL-12 can induce the expression of cutaneous lymphocyte antigen (CLA) on lymphocytes [7, 8]. This homing receptor is responsible for directing lymphocyte trafficking into the skin. Regarding T cell differentiation, IL-12 also has strong antagonist actions on Th2 pathway polarization [9] and the class switch towards immunoglobulin (Ig) E. IL-12 has also been described for its inhibitory impact on retinoic acid receptor-related orphan receptor-γT (RORγT), a key transcription factor for Th17 polarization. As a result, IL-12 can act on differentiating human Th17 cells to switch them to more IFNγ production.

### Main Actions of Importance for Psoriasis

**Fig. 1:**
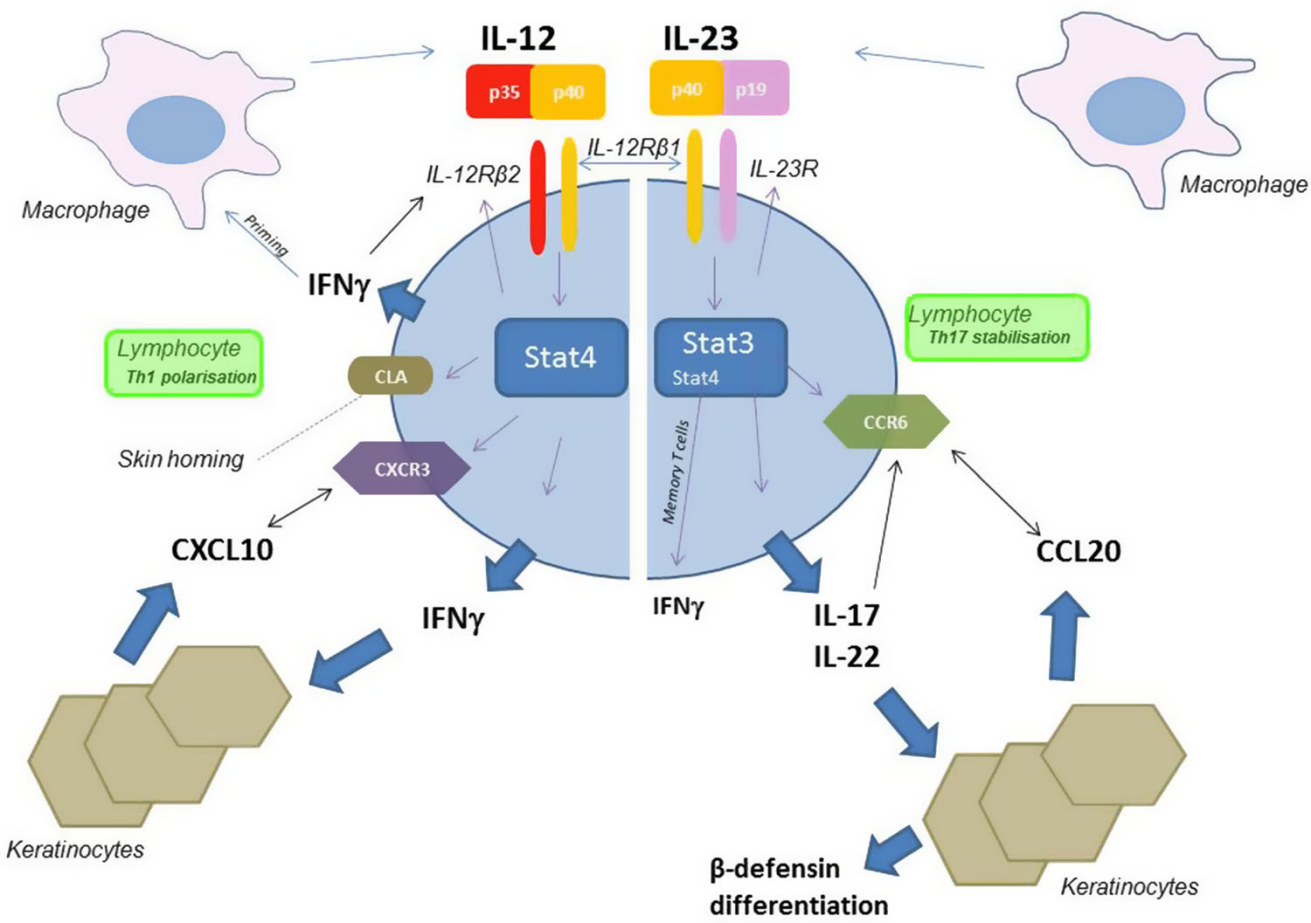
Schematic, simplified overview of IL-12/IL-23 dependent action on molecules involved in psoriatic inflammation. Only positive/activating pathways are depicted. Both IL-12 and IL-23 are produced by activated APCs including macrophages. Upon receptor ligation, these heterodimeric cytokines activate the Stat4/3 pathways ultimately resulting in upregulation of cell surface receptors and secretion of cytokines. Of importance, type 17 cells, which are dependent on IL-23 stimulation, express high levels of CCR6 which enables the cell to follow a chemokine gradient build by CCL20 which is produced by IL-17/IL-22 stimulated keratinocytes. Thus type 17 cells will home into CCL20 rich tissues. On the other hand, IL-12, via activation of Stat4, acts on expression of the skin homing receptor CLA but also CXCR3 which interacts with the chemokines CXCL9, 10, 11 which are all highly expressed by keratinocytes which have been exposed to IFNs. IFNγ is one of the strongest priming signal for APC to induce the production of the IL-12 family members IL-12 and IL-23. Negative regulatory feedback actions are not depicted in this figure. However, there is significant negative cross-regulation between type 1 and type 17 cells. Blocking the p40 subunit of both IL-12 and IL-23 could therefore result in “paradoxical” effects where this negative regulatory influence plays an important role in disease pathology. *APC* Antigen presenting cell, *CLA* Cutaneous lymphocyte antigen, *IFN* Interferon, *IL* Interleukin

IL-12 is key for the production of INFγ which is one of the strongest activators of keratinocyte proinflammatory responses (Fig. 1). IFNγ induces the production of CXCL10 in keratinocytes, which attracts even more CXCR3 + IFNγ producing T cells. Similar mechanisms are in place on the level of the synovium. The proinflammatory properties of both IL-12 and IFNγ can be enhanced by TNFα. IL-12 can furthermore play a role in the homing of lymphocytes into the skin by virtue of its action on CLA expression.

IL-23 is also mainly produced by activated APCs (macrophages, DCs) [10]. IL-23 was only described a number of years after IL-12 and many of the early studies which measured IL-12p40 did not distinguish between IL-23 and IL-12 functional effects. All initial data on IL-23 highlighted this cytokine as inducer of IFNγ production. Indeed, human IL-23 induces the proliferation and the production of IFNγ by memory T cells. However, unlike IL-12 it does not act on Th1 polarization and does not support naïve T cells to develop to IFNγ producers. Different from IL-12, IL-23 is critical for activation, survival, and expansion of type 17 cells [10–12]. RORγT positive T cells, NKT cells and ILC which can produce IL-17 are termed type 17 cells. IL-23 stabilizes IL-17 expression but, unlike IL-12’s action on Th1 cells, does not act as differentiation factor for Th17 polarization. IL-23 induces a proinflammatory signature that includes IL-17, TNFα, CCL20, IL1R1 and IL-23R. By acting on (1) IL-23R expression which increases the cellular sensitivity to IL-23, (2) on CCL20, a chemokine which directs the movement of CCR6+ IL-17 producers, and (3) IL-17 production, a strong positive feedback loop supporting “type 17 inflammation” is created which plays an important role in chronic psoriatic inflammation. IL-1 and IL-23 act in synergy to induce local tissue inflammation and IL-23 increases the cellular sensitivity to IL-1 by acting on its receptor expression.

### Main Actions of Importance for Psoriasis

Actions of IL-23 will ultimately lead to IL-17 production and support the survival and via CCL20 the recruitment of type 17 cells (Fig. 1). Those cells also produce IL-22. Both IL-22 and IL-17 directly activate keratinocytes but also synovial cells. IL-17 and TNF show synergistic proinflammatory functions. IL-17 and IL-22 are responsible for high production of antimicrobial peptides (including β defensins) which play a chemotactic and proinflammatory role in psoriasis pathogenesis. Furthermore, IL-22 (and related IL-20 subfamily members) are key to the changes seen in keratinocyte differentiation and proliferation which are so characteristic for the psoriatic phenotype.

## Main Function of IL-12/23P40 Blockade in Psoriasis

This therapeutic intervention can act on many different levels as highlighted above. Reducing the proinflammatory actions of the effector cytokines IFNγ and IL-17/IL-22 seems key. However, there is also some counter-regulation between type 1 and type 17 cells. Depending to the “molecular” subtype of psoriasis or the disease to be treated, breaking this counter regulatory balance could, to some extent, lead to “weakening” of the anti-inflammatory action of the inhibitor. On the other hand, in theory, higher availability of p35 subunit (although widely expressed) could lead to increase in the regulatory IL-12 family member IL-35 which is a p35/EBI3 heterodimer; however, this remains to be shown.

### IL12/23 Inhibitors in Other Dermatologic Diseases

Sarcoidosis is a disease with high IL-12 activity. Both the p40 subunit as well as the expression of the high affinity IL-12Rβ2 has been found increased in this disease. While there are reports on successful treatment of sarcoidosis with UST there is also a case report suggesting a paradoxical sarcoidosis promoting effect under p40 inhibition, similar to what has been reported for TNF blockade [13]. A recent study on skin and lung sarcoidosis patients suggests that blockage of TNF may result in more favorable therapeutic effects for the skin than that of p40 blockade for the time period observed [14]. Case reports suggest that UST could be of benefit in therapy resistant Pyoderma gangrenosum [15, 16], hidradenitis suppurativa [17, 18], SAPHO syndrome [19], and pityriasis rubra pilaris [20–23].

## Clinical Studies in Psoriasis

In January 2009, UST (CNTO 1275, Stelara; Janssen Cilag) was granted marketing authorization by the European Commission for the treatment of moderate to severe chronic plaque psoriasis in adults who failed to respond to, who have a contraindication to or who are intolerant of systemic oral immunosuppressants. Unlike the other biological anti-psoriatic agents already brought to market, which all targeted TNFα, UST was the first-in-class anti-IL agent for psoriasis, representing an important milestone in rational drug design. UST is a fully human IgG1κ monoclonal antibody that inhibits IL-12 and IL-23 activity by binding with high affinity and specificity to their shared p40 subunit. IL-12/23 bioactivity is thus inhibited by preventing their binding to IL-12 receptor β1 (IL-12Rβ1) on the surface of immune cells.

### Phase I and II Clinical Trials

UST’s therapeutic potential was apparent in early phase I studies, with reductions in lesional gene expression of IL-12p40, IL-23p19 and other inflammatory cytokines as early as two weeks post treatment [24–26]. The drug was well tolerated and appeared to have low immunogenic potential. In some patients, a single intravenous or subcutaneous dose resulted in a rapid and marked clinical response that was sustained for 16–24 weeks. In an initial phase II randomized trial [27], 320 patients were allocated to one of five groups, receiving placebo or one of four doses of UST (45 or 90 mg once only, or 45 or 90 mg weekly for 4 weeks). At week 16, patients treated with UST with a physician’s global assessment (PGA) of three or more received an additional injection of their initial dose. At week 20, those in the placebo arm received a single 90 mg dose. The primary endpoint of Psoriasis Area and Severity Index (PASI) 75 response at week 12 showed statistical significance for all active treatment groups (51.6% for 45 mg once only, 59.4% for 90 mg once only, 67.2% for four doses of 45 mg weekly, 81.3% for four doses of 90 mg weekly), compared with 1.6% in the placebo group. Clinical responses were maintained out to week 24 before deterioration and were supported by substantial improvements in the Dermatology Life Quality Index (DLQI). Serious adverse events were not statistically higher in any group.

### Phase III Clinical Trials

Following on from the promise shown in the phase II study, the safety and efficacy of UST were further assessed in three large phase III clinical trials involving 2,899 adult patients with moderate to severe psoriasis (PASI >12, PGA ≥3 or 10% body surface area involvement) of at least 6 months duration and who were candidates for systemic immunosuppression or phototherapy. Run in parallel, both PHOENIX I (ClinicalTrials.gov #NCT00267969) [28] and PHOENIX II (ClinicalTrials.gov #NCT00307437) [29] were multicenter, randomized, double-blind, placebo-controlled trials with similar objectives and methods and a primary endpoint of PASI 75 response from baseline at week 12. The same primary endpoint was selected for the third, the ACCEPT (ClinicalTrials.gov #NCT00454584) trial, but differed in that it compared UST with etanercept in place of placebo [30].

PHOENIX I, a 76 week study, involved 766 patients, 53% of which were either non-responsive to, intolerant of or had a contraindication to other systemic therapy [28]. Participants were initially randomized (1:1:1) to placebo or active treatment with UST at either 45 mg or 90 mg subcutaneously at week 0, 4, and then every 12 weeks thereafter. Baseline randomization was stratified by study site, weight (≤90 or >90 mg) and the number of systemic therapies to which the patient had had an inadequate response, intolerance or contraindication (<3 or ≥3).

Patients in the active treatment group who achieved a PASI 75 response at both weeks 28 and 40 were re-randomized at week 40 to maintenance UST (same dose as initial stage) or withdrawal from treatment (placebo administered) until loss of response. Randomization at week 40 was also based on study site and patient weight. Patients in this group who had achieved a partial (PASI 50–PASI 74) response at week 40 were adjusted to a dosing interval of every 8 weeks. Patients randomized to receive placebo at week 0 and week 4 crossed over to receive UST (45 or 90 mg) at weeks 12 and 16, followed by dosing every 12 weeks thereafter. This study design allowed not only comparison of UST against placebo, but also long-term efficacy, duration of therapeutic effect after drug withdrawal and possible dose escalation in partial responders [28].

At the primary endpoint (week 12), 67.1% of those receiving the 45 mg dose and 66.4% of those receiving 90 mg achieved PASI 75, compared to 3.1% of the placebo group (*P* < 0.0001). Improvement was rapid, with many in the active treatment groups, regardless of dose, achieving PASI 50 by week 2. Maximum efficacy was observed at week 24 for both dosing regimens (76.1 and 85.0% PASI 75 response for 45 and 90 mg, respectively), with similar findings in the group initially assigned to placebo after crossing over to active treatment at week 12 [28].

After re-randomization at week 40, either maintenance therapy or withdrawal, preservation of PASI 75 was significantly greater in those receiving continuous UST therapy (84% at week 76) compared with the treatment withdrawal group (19% at week 76). In the latter, PASI scores began to deteriorate by week 44 (16 weeks after last injection), and accelerated after week 52. Withdrawn patients were re-treated with their initial dose after their PASI 50 response was lost. A total of 195 patients needed to restart therapy and 85.6% regained their PASI 75 after 12 weeks of re-treatment [28].

Expanding on the initial PHOENIX I trial data reported at week 76, all patients were subsequently followed to week 244 (5 years) to assess longer-term safety and efficacy [31, 32]. Overall, 68.7% (*n* = 517) of the initial overall population of 753 (who had received at least one dose of UST in PHOENIX I) were evaluated. Initial clinical responses were generally maintained through week 244 (PASI 75: 63.4 and 72.0%; PASI 90: 39.7 and 49.0%; PASI 100: 21.6 and 26.4% for patients receiving 45 or 90 mg, respectively) [31]. At week 264, analysis of 8998 patient years of follow-up demonstrated event rates (per 100 patient years; 45 and 90 mg, respectively) for all adverse events (242.6, 225.3), serious adverse events (7.0, 7.2), serious infections (0.98, 1.19), non-melanoma skin cancers, or NMSCs (0.56, 0.36), other malignancies (0.59, 0.61), and major adverse cardiovascular events, or MACE (0.56, 0.36), that were comparable between the two dosing groups. No increasing trend in any adverse events was seen over time, and the rates of overall mortality and other malignancies were comparable with the general population of the United States [32].

Nail involvement may be present in up to 80% of patients with psoriatic disease and is notoriously difficult to treat, leading to high psychosocial embarrassment [33] and in severe cases, functional limitation [34]. Improvements in fingernail psoriasis were assessed in the PHOENIX I cohort using the Nail Psoriasis Severity Index (NAPSI) on a target fingernail in addition to a nail PGA and assessment of the mean number of nails involved [35]. Of the 766 randomized, 545 had nail psoriasis. By week 24, the percentage improvement in NAPSI from baseline was 46.5 and 48.7% for UST 45 and 90 mg, respectively. Improvements in the less sensitive nail PGA scores were generally not observed in the overall nail psoriasis cohort at week 12; however, substantial improvements were noted at week 24, with the majority of patients with a PGA ≥3 at baseline achieving improvement by at least one point. In the 45 and 90 mg groups, 77.0 and 75.0%, respectively, of patients with moderate nail disease (PGA 3) improved to mild (PGA 2) or clear (PGA 1) by week 24.

The second large phase III clinical trial, PHOENIX II, recruited 1,230 patients and 61% were either non-responsive to, intolerant of or had a contraindication to other systemic therapy [29]. Like PHOENIX I, patients were randomized to one of three arms; 45 mg or 90 mg subcutaneously at week 0, 4 and every 12 weeks, or placebo at weeks 0 and 4 and then crossover to active therapy (randomized 1:1 to either 45 or 90 mg) at week 12 (with loading doses at week 12 and 16, followed by injections every 12 weeks thereafter). At week 28, patients were deemed responders (PASI 75 response achieved), partial responders (PASI 50–74) or non-responders (PASI < 50). Responders continued treatment at the same dose every 12 weeks, non-responders discontinued therapy, and partial responders were re-randomized to either continue their current regimen or reduce their dosing interval to every 8 weeks. Stratification was as described for PHOENIX I. The partial responder group permitted analysis of the number of visits between weeks 28 and 52 where PASI 75 was achieved for the two different dosing schedules.

At week 12 (primary endpoint), 66.7% of patients receiving UST 45 mg and 75.7% of patients receiving 90 mg every 12 weeks achieved PASI 75 (*P* < 0.0001), compared with 3.7% of participant receiving placebo. Maximum efficacy was seen around week 20 for both doses (PASI 75 in 74.9 and 83.5% for 45 and 90 mg, respectively), with similar outcomes seen in the placebo group after crossing over to active therapy. In those who achieved PASI 75 by week 28, the improvement was maintained until the end of the study (week 52). In all, the median clinical response at the end of the study was PASI 95 for those in the 45 mg group and PASI 96 for those in the 90 mg group [29].

Partial responders accounted for 22.7% of those receiving 45 mg every 12 weeks, and 15.8% of those receiving 90 mg. At baseline, compared to responders, these individuals were of a greater body weight, had more severe PGA scores, a longer duration of psoriasis, a greater incidence of PsA, a higher failure rate with previous systemic immunosuppressants and lower serum drug levels at week 28. For those receiving 90 mg, a reduction in the dosing interval from 12 to 8 weeks did equate to a greater number of visits where a PASI 75 response was achieved, but this was not the case for those receiving 45 mg [29].

In terms of safety, both PHOENIX I and II reported similar outcomes during the placebo-controlled phase. Adverse events occurred in 278 (54.5%) of the 510 patients receiving UST in PHOENIX I [28] and 414 (50.5%) of 820 patients in PHOENIX II [29]. This is compared with 48.2 and 49.8% in their respective placebo groups. Serious adverse events occurred in similar proportions in both trials and with similar low frequencies between the UST and placebo treated arms (1.2% UST vs. 0.8% placebo in PHOENIX I; 1.6% UST vs. 2.0% placebo in PHOENIX II). In PHOENIX I, the pattern of adverse events was much the same in the placebo crossover and randomized withdrawal phases as it was in the placebo-controlled phase [28]. Rates of antibody formation to UST were found in 5.1% of patients by the end of week 76 (PHOENIX I) and 5.4% of patients by the end of week 52 (PHOENIX II), and in both trials, these were mostly of low titer.

The ACCEPT phase III clinical trial differed from the PHOENIX trials in that the safety and efficacy of UST were compared with an active comparator (etanercept) rather than placebo [30]. In this 64 week trial, 903 patients were randomized (3:5:5 ratio) to receive subcutaneous injections of UST (45 or 90 mg) at weeks 0 and 4, or etanercept (50 mg) twice weekly for 12 weeks. Randomization was stratified according to study site and baseline weight (<90 or ≥90 mg). Patients were aware of their treatment, but study assessors remained blinded. At week 12, patients in the etanercept group who did not respond (classified as moderate, marked or severe psoriasis on the PGA) were given 90 mg UST at weeks 16 and 20, and those who did not respond in the UST group were given one further additional dose of UST at week 16. For those who did respond (classified as clear, minimal, or mild) at week 12, treatment was withdrawn. If psoriasis recurred and was graded moderate, marked or severe, patients were retreated with UST, regardless of initial therapy.

At week 12, 67.5 and 73.8% of patients receiving 45 and 90 mg UST respectively achieved PASI 75, compared with 56.8% of those receiving etanercept (*P* = 0.01 and *P* < 0.001, respectively), and the time to improvement was more rapid in those treated with UST. PASI 90 responses were achieved in 36.4% of patients receiving 45 mg UST, 44.7% of patients receiving 90 mg UST and 23.1% of patients receiving etanercept (*P* < 0.001 for both). Amongst those patients who were deemed non-responders to etanercept, 48.9% achieved PASI 75 and 23.4% achieved PASI 90 12 weeks after crossing over to UST. For those who were graded as responders at week 12 and had therapy withdrawn, the median time to recurrence was 14.4 weeks (45 mg UST), 18.1 weeks (90 mg UST) or 7.3 weeks (etanercept). Of the 633 patients who were retreated after re-emergence of moderate to severe psoriasis, 534 were classed as having mild, minimal or no psoriasis within 12 weeks [30].

Adverse events occurred with similar frequency across all treatment groups, with at least one event in 70.0% of etanercept-treated participants, 66.0% in the 45 mg UST group and 69.2% in those receiving 90 mg UST. Most adverse events were classed as minor, with only 12 patients (4 in each group) from the 903 recruited having a major event. Overall, discontinuation of therapy was necessary in similar proportions, ranging from 1.2 to 2.3%. A noticeable discrepancy was seen in injection site reactions (24.8% of patients who received etanercept as compared with 4.3% (45 mg) and 3.7% (90 mg) of patients receiving UST), although it is worth acknowledging the higher number of injections necessitated by the dosing schedule of the etanercept arm. Through to week 12, infections occurred at comparable rates in the three treatment groups (29.1, 30.6, and 29.7% in the groups that received etanercept, 45 mg UST, and 90 mg UST, respectively) and this was relatively consistent to the end of the trial. NMSCs occurred only in patients treated with UST but at low numbers (three by week 12 and a further nine by week 64). Quality of life indices were not recorded in the ACCEPT trial [30].

Several other smaller phase III clinical trials have assessed the safety and efficacy of UST in non-western populations and found similar clinical responses. In the PEARL trial, 121 Taiwanese and Korean patients with moderate-to-severe psoriasis were enrolled into a 36 week, multicenter, double-blind, placebo-controlled trial to receive UST 45 mg at week 0, 4 and 16, or placebo at week 0 and 4, followed by UST at week 12 and 16 [36]. At the primary endpoint (week 12), PASI 75 was achieved by 67.2% in the UST-treated group, and 5.0% in the placebo arm (*P* < 0.001). Efficacy was maintained through to week 28 in the UST group. Adverse events were similar between the groups, with exception of abnormal hepatic function, which was related to concomitant isoniazid treatment for latent tuberculosis. No deaths, malignancies, or MACE were reported. An identical study design was employed in a 72 week phase II/III clinical trial involving 158 Japanese patients, with the addition of 90 mg UST arm [37]. At week 12, 59.4% and 67.7% of UST 45 mg and 90 mg treated patients achieved PASI 75, compared with 6.5% in the placebo group (*P* < 0.0001). By week 12, rates of infections were comparable amongst the groups (UST 45 mg, 20.3%; 90 mg, 24.2%; placebo, 18.8%), and only single cases of serious infections and non-cutaneous malignancies were recorded, both occurring in the 90 mg ustekinumab group. There were no reports of NMSC. Through to week 72, similar rates and types of adverse reactions and serious adverse were reported between the 45 and 90 mg ustekinumab-treated groups.

### Factors Influencing Clinical Response to UST

HLA-Cw06 has long been established as the most potent psoriasis susceptibility gene. However, latterly, observations have suggested that this genetic polymorphism could serve as a pharmacogenetic marker to predict clinical response to immunomodulatory agents including UST [38]. Talamonti et al. [39] observed a statistically significant increased response to UST in HLA-Cw06–positive patients (PASI 75 response at week 12: 96.4 vs. 65.2% in HLA-Cw06–negative individuals). The time to response was also faster, with 89.3% of HLA-Cw06–positive patients reaching PASI 50 at week 4 (after one single dose) compared to 60.9% of HAL-Cw06–negative patients. No significant association was found between clinical response and the other psoriatic genetic markers studied (TNFAIP3rs610604 polymorphism and LCE3B/3C gene deletion) [39]. Genetic susceptibility to psoriasis can vary between races, although Chiu et al. [40] replicated the Italian study in Chinese patients with psoriasis.

In addition to genetic factors, obesity has been recognized as an important factor related to both the incidence and severity of psoriasis [41]. Obesity can induce an overproduction of multiple proinflammatory cytokines in adipose tissue, including TNFα, IL-6 and IL-8, all of which are implemented in the pathogenesis of psoriasis [42]. Lebwohl et al. [43] evaluated the effect of weight on response to UST in patients enrolled into the PHOENIX I and II trials and found that those with a body mass greater than 100 kg had a reduced efficacy to UST. The proportion of patients with a body mass ≤100 kg achieving PASI 75 was 76.9%, compared to 54.6% in those weighing >100 kg at the 45 mg dose, and 80.8% (≤100 kg) compared to 74.2% (>100 kg) at the 90 mg dose. Serum drug concentrations were also affected by weight, and together these findings provided the rationale for the higher dose subsequently licensed for patients weighing more than 100 kg [43].

### Quality of Life Response

In PHOENIX I, more than 97% of patients had a score of 1 or more on the DLQI at baseline, and the average score was greater than 10 out of a possible maximum of 30, indicating a significant impact on patients’ quality of life [44]. Significantly greater proportions of patients receiving UST 45 mg and 90 mg achieved a normalization of DLQI (≤1) compared with placebo (53.2, 52.4, 6.0%, respectively, both *p* < 0.001) at week 12. The SF-36 questionnaire revealed similarly impressive results for both the physical (45 mg, 23.1%; 90 mg, 33.7%; placebo, 15.6%) and mental (45 mg, 25.5%; 90 mg, 31.3%; placebo, 14.8%) component scores by week 12 (*P* < 0.001). The greatest improvements were found in the bodily pain and social functioning domains. These quality of life improvements were sustained with maintenance UST therapy at one year.

In the PHOENIX II trial, the Hospital and Anxiety Depression Scale (HADS) replaced the SF-36, alongside the DLQI [45]. At baseline, a high psychological impact of disease was apparent, with 40.3% in the group receiving UST 45 mg, and 26.7% receiving 90 mg, reporting symptoms of anxiety and depression, and 54.6% reporting a DLQI ≥10. By week 12, the absolute mean (±SD) reduction in DLQI was by 9.3 points (±7.1) in the 45 mg group, 10.0 (±6.7) points in the 90 mg group, compared with −0.5 (±5.7) in the placebo arm. The proportion of patients with baseline symptoms of mild to severe anxiety (as assessed by HADS-A) decreased from 38.2 to 25.7% by week 12 in the UST 45 mg group and from 41.0 to 27.1% in the UST 90 mg group (*P* < 0.001 vs. placebo), representing a combined relative reduction of 34% from baseline (compared with a 1.4% increase in the placebo group). The prevalence of baseline symptoms of mild to moderate depression (as assessed by HADS-D) decreased from 24.7 to 12.8% by week 12 in the UST 45 mg group and from 31.1 to 12.5% in the 90 mg group (*P* < 0.001 vs. placebo), representing a relative reduction of 55% from baseline (compared with an increase of 10% in the placebo group).

Sexual difficulties were specifically analyzed from the DLQI data collated in both PHOENIX I and II [46]. Impaired sexual function was recorded if any patient scored ‘very much’ or ‘a lot’ for question 9 of the DLQI. 27.1% of women and 20.8% of men reported impaired sexual function at baseline, and this was significantly associated with increased psoriasis severity. At week 12, the overall proportion of patients with sexual difficulties decreased from 22.6 to 2.7%, compared to no change in the placebo arm (*P* < 0.001). Patients with a greater mean improvement in PASI score experienced a greater reduction in sexual difficulties caused by psoriasis.

## Clinical Studies IN PsA

For UST, in PsA, the main phase III studies are PSUMMIT-1 (ClinicalTrials.gov #NCT01009086) [47] and PSUMMIT-2 (ClinicalTrials.gov #NCT01077362) [48]. In PSUMMIT-1, 615 patients with active PsA, stratified by weight and methotrexate (MTX) use, were randomized to placebo, UST 45 mg or UST 90 mg (injections were given at weeks 0, 4 and every 12 weeks thereafter. At week 12 patients with an inadequate response (<5% improvement in tender and swollen joint counts) could escalate (placebo to UST 45 mg, UST 45 mg to UST 90 mg, but no escalation if the patient was already taking UST 90 mg). The primary outcome measure was the American College of Rheumatology (ACR) 20 rate at week 24 (Table 1). Significant differences between groups, in favor of UST, were seen (placebo 22.8%, UST 45 mg 42.4%, UST 90 mg 49.5%). As with TNF inhibitors (TNFi), the concomitant use of MTX did not appear to make any difference to the efficacy of this drug (combined UST groups: ACR20 with MTX 44.5%, without 47.5%). The kinetics of the ACR response suggested that a peak was reached at week 28. As for the other manifestations of psoriatic disease, skin, dactylitis and enthesitis showed significant improvement with both doses of UST and, in cases with spondylitis, significant improvement in Bath Ankylosing Spondylitis Disease Activity Index (BASDAI) scores. At week 16 adverse events were similar between placebo and active drug groups with nasopharyngitis, upper respiratory infections and headache the main adverse events with UST. Over 52 weeks, 4 serious infections were reported. Of note, three major cardiovascular events occurred in the UST groups in the first 30 weeks of treatment.

**Table 1:** ACR20 rates for ustekinumab in phase III studies

	PSUMMIT-1				PSUMMIT-2			
	Week 12*	Week 24			Week 24			
	ACR20	ACR20	ACR50	ACR70	ACR20	ACR50	ACR70	ACR20 (TNF_IR_)
Placebo	21	23	9	2	23	7	3	15
UST45 mg	41	42	25	12	44	18	7	37
UST90 mg	41	50	28	14	44	23	9	35

All figures are (rounded) percentages

*ACR* American College of Rheumatology

* Taken from figure 2A in McInnes et al. [47]

Patients in PSUMMIT-1 were TNFi naïve. PSUMMIT-2 addressed the issue of previous TNFi exposure. Although a similar design was employed (same criteria for active disease, randomization and early escape, stratification for weight and MTX use) just over half of the 300 patients recruited had prior TNFi exposure. Of these TNFi experienced patients the majority had used more than one agent and 70% had discontinued the drug because of inadequate response. In PSUMMIT-2 the primary end-point of superiority over placebo at week 24 was reached despite the more challenging patient population and the smaller sample size (Table 1). As expected response rates in those patients who were TNFi experienced were inferior to TNFi-naïve patients. Of interest, results were independent of MTX usage and, generally, weight, although response was not as good in patients over 100 kg in the UST 90 mg group. As with PSUMMIT-1 improvement was seen in skin, enthesitis, BASDAI in patients with spinal inflammation, fatigue, and function but not for dactylitis. No major MACE events were seen up to week 16 but up to week 60 the myocardial infarction rate was 0.74/100 patient years.

In order to achieve a sufficient sample size, structural progression analyses were pre-specified for a pooled analysis of PSUMMIT-1 and PSUMMIT-2 patients [49]. Thus, 927 patients were available with a missing data rate of about 10% overall (there were more patients who were TNFi experienced in placebo arm with missing data than in other groups). A significant reduction in radiographic progression at 24 weeks was found with changes in the modified Sharp van der Heijde score (mSvdH) of 1 in the placebo arm and 0.4 in both UST arms. Median scores for change were, however, low over the whole cohort. At 24 weeks the percentage of patients with a radiographic change score which was less than the smallest detectable difference was 83.8% in the placebo arm, 91.7% in the UST 45 mg arm and 91.9% in the UST 90 mg arm. Over the 52 week observation period these rates of progression remained stable. The mSvdH score does not measure new bone formation, so no information was available on this important radiographic feature. Cases with osteolysis and ‘pencil in cup’ were noted separately and were also infrequent and stable over 52 weeks.

To date, the only other available data on UST comes from meeting abstracts. Safety data from pooled psoriasis and PsA studies were reported at the ACR meeting in 2013 [50] and 2014 [51]. No worrying signals for serious infection or malignancy were found but MACE events once more appeared higher in the UST treated patients, although confidence intervals (CI) did overlap with the placebo rate (placebo: rate of events per 100 patient years of exposure (95% CI): 0 (0–1.69); UST 1.23 (1.40–2.87)). In 2014, pooled data over a two-year period did not support an increase in MACE events in either dose (45 or 90 mg) of UST [52]. Of interest, and as a complement to the data in the PSUMMIT studies on patients with spondylitis, Poddubnyy et al. [53] reported a small, open-label, proof of concept study of UST in ankylosing spondylitis. In this study, 20 patients with AS were given three doses of UST at weeks 0, 4, and 16 and assessments were made at week 24. Significant improvements in the traditional AS outcome measures were seen with 65% of patients achieving an Assessment of SpondyloArthritis international Society (ASAS) 40 response rate. Unlike with TNFi drugs, no change in CRP was seen overall, but the CRP was significantly reduced in the responders. In 2014, in abstract format, concomitant reduction in inflammation identified on magnetic resonance imaging was also demonstrated in this cohort [54].

## Discussion

UST is a new class of drug specifically targeting the IL-12/23 axis and is effective in psoriasis and PsA. What is the likely use of this drug in clinical practice? It is worth considering the current treatment algorithms in use in this disease. Psoriasis and PsA will be considered separately and then as a combined approach.

From the data available UST is a valuable addition to psoriasis treatment, providing a potent biologic alternative to the TNFi class of drugs. The place of UST is similar to TNFi and the decision to start UST as opposed to TNFi may depend on such factors as contraindications to TNFi, cost, and patient preference, given the alternative dosing schedules of this drug. There are as yet no data on the safety profile of UST with ultraviolet therapy but it might be assumed that their safety profile would be favorable when compared to drugs such as TNFi.

When a new drug works well for psoriasis the most important question concerns its efficacy against the articular part of the disease. From a patient point of view, a drug that works against all aspects of the disease is ideal, and if topical therapy can be avoided, so much the better. TNF inhibitors fulfil this role well and remain the yardstick by which other drugs are measured. However, not all drugs active against psoriasis work for the joints. The prime example was efaluzimab, now withdrawn, which may have induced cases of PsA in patients with psoriasis [55]. UST is clearly effective in PsA: not only the articular manifestations but those other features so characteristic of the disease: enthesitis, dactylitis, and spondylitis. At this moment TNFi drugs set the benchmark of response in PsA and, on the evidence from the PSUMMIT trials UST is probably not quite as efficacious as TNFi for the articular manifestations. However, only head-to-head data will be able to confirm this observation given the possible disparity of patient populations across the studies. Nevertheless, UST, like TNFi is effective for all aspects of the disease, skin and musculoskeletal and, from a patient point of view this is an attractive feature.

The kinetics of response may be of some concern. If a rapid response of skin and joints is required then the physician is more likely to recommend a TNFi, or one of the IL-17 inhibitors. On the other hand, if there has been TNFi failure, then UST may be an attractive option, given the results of the PSUMMIT-2 study. More data is needed on the response to UST according to primary or secondary non-response to TNFi—it is likely these data are available from the PSUMMIT-2 study and will no doubt appear in due course. The 52 week results also suggest that alternative dosing schedules may be required for articular and other musculoskeletal manifestations, possibly with a shorter dosing interval.

UST will have a place in treatment when TNFi are contra-indicated (currently with a history of demyelinating disorder, active tuberculosis, or a recent malignancy) although it must be emphasized that we still do not know the safety of UST in these situations. A doubt still remains about MACE events with UST and only long term surveillance using registry data will be able to illuminate this and other safety concerns.

Should UST be co-prescribed with MTX? The studies so far do not indicate any enhancement of efficacy with MTX use but, as with TNFi, MTX may prolong the effective period of this drug, although the rate of antibody formation to UST seems low. However, many patients find the higher doses of MTX unacceptable so it would be prudent to use a low maintenance dose of 10–15 mg.

## Conclusion

UST provides another weapon in the physicians armory, with a new target. Cost issues will be equivalent to TNFi and other biologics so its place in the treatment algorithm of psoriasis and PsA will evolve over time and with continued use. The different mode of action will offer a treatment alternative to TNFi failures but the rather slow onset of action may be a problem for some patients and their physicians.

## Electronic supplementary material

Below is the link to the electronic supplementary material.

Supplementary material 1 (PDF 185 kb)
