# Supplementary material for: Ustekinumab in the Treatment of Psoriasis and Psoriatic Arthritis
Source: Rheumatol Ther. 2015 Mar 17;2(1):1–16. doi: 10.1007/s40744-015-0010-2 (PMC4883251; doi:10.1007/s40744-015-0010-2)
Supplement: Supplementary file 1 — Supplementary material 1 (PDF 185 kb) [file 40744_2015_10_MOESM1_ESM.pdf]

- Not all patients with psoriatic disease respond to tumor necrosis factor (TNF) inhibitors, indicating the presence of other important inflammatory pathways in this disease.
- The production of interleukin (IL)-12 and IL-23 are important in psoriatic disease because of their downstream effects.
- Targeting IL-12/23 with drugs such as ustekinumab provides an alternative treatment approach where TNF inhibitors have failed or never been effective.
- Clinical studies support the use of ustekinumab in both psoriasis and psoriatic arthritis, with Psoriasis Area Severity Index (PASI) 75 rates in excess of 80% and American College of Rheumatology (ACR) 20 rates of 50% 24 weeks after the start of treatment.
- Adverse event rates are similar to the other biologic drugs used in psoriatic disease.

This summary slide represents the opinions of the authors. No funding or sponsorship was received for this study or publication of this article. For a full list of acknowledgments and conflicts of interest for all authors of this article, please see the full text online. Copyright © The Author(s) 2015. Creative Commons Attribution Noncommercial License (CC BY-NC).
